# Cognitively Impaired Old Mice Display Correlated Reduction in Cortical NMDA Receptor and Complex IV

**DOI:** 10.1093/geroni/igaa057.388

**Published:** 2020-12-16

**Authors:** Ramkumar Thiyagarajan, Yonas Redae, Kenneth Seldeen, Bruce Troen

**Affiliations:** 1 University at Buffalo, Buffalo, New York, United States; 2 University of Buffalo, Buffalo, New York, United States

## Abstract

Cognitive decline in older adults represents a major challenge since cognitive impairment is found in 10% of those ≥ 65 and 50% ≥ 85. Thus it is increasingly important to understand the impact of aging on cognitive health. We performed a battery of tests to assess cognition in 6 month-old (n=12) and 24 month-old (n=8) C57BL/6J mice, equivalent to 30 and 70 year old humans, respectively, and also assessed protein markers in cortex for mitochondrial health and cognition. We found that aged mice displayed fewer spontaneous alternations in the T maze test (p=0.034) and lower recognition of novel objects (p=0.022). In addition, aged mice showed prolonged escape time in the Barnes maze (p=0.035), all of which taken together suggest reduced capacity for learning and recall. Aged mice also exhibited diminished nest building (p<0.001), revealing an impaired functional capacity analogous to the instrumental activities of daily living (IADL) geriatric assessment. We found reduced mitochondrial complex IV expression in the cortices of aged mice concomitant with less expression of N-Methyl D-Aspartate (NMDA) receptor subunits 1, 2A and 2B. The cortices from old mice also exhibited greater expression of immature brain derived neurotrophic factor (pro-BDNF). The alterations in NMDA receptors and pro BDNF are consistent with memory impairment and greater neuronal cell death. Therefore, aged mice exhibit significantly reduced recall and learning ability alongside marked alterations in mitochondrial complex, NMDA receptor, and pro-BDNF expression. Studies are underway to assess whether these molecular changes are responsible for the cognitive declines with aging.

